# Comparison of Granular Bone Grafts and Transverse Process Bone Grafts for Single-Segmental Thoracic Tuberculosis: A Retrospective Single-Center Comparative Study

**DOI:** 10.3389/fsurg.2021.602513

**Published:** 2021-05-14

**Authors:** Xing Du, Yunsheng Ou, Yong Zhu, Wei Luo, Guanyin Jiang, Dianming Jiang

**Affiliations:** Department of Orthopedics, The First Affiliated Hospital of Chongqing Medical University, Chongqing, China

**Keywords:** posterior debridement, internal fixation, granular bone graft, transverse process bone graft, thoracic tuberculosis

## Abstract

**Background:** To compare the clinical efficacy of granular bone grafts and transverse process bone grafts for single-segmental thoracic tuberculosis (TB).

**Methods:** The clinical records of 52 patients who were diagnosed with single-segmental thoracic TB and treated by one stage posterior debridement, bone graft fusion, and internal fixation in our department from 2015 to 2018 were retrospectively analyzed. Among them, 25 cases were in the granular bone graft group and 27 cases in the transverse processes bone graft group. Outcomes including the visual analog scale (VAS), erythrocyte sedimentation rate (ESR), C-reactive protein (CRP), neurological function, operative time, operative blood loss, hospital stay, Cobb angle, bone graft fusion time, and postoperative complications were all recorded and analyzed.

**Results:** There were no significant differences in operative time, operative blood loss, and hospital stay between the two groups (*P* > 0.05). With an average follow-up of 18–33 months, all patients in the two groups showed significant improvement in VAS score, ESR, CRP, and neurological function compared with preoperative measurements (*P* < 0.05), however, no significant differences were found for the last follow-up (*P* > 0.05). The two groups showed similar Cobb angle correction (*P* > 0.05), but the granular bone graft group had a larger Cobb angle loss than the transverse processes bone graft group (*P* < 0.05). The bone graft fusion time of the granular bone graft group was shorter than that of the transverse processes bone graft group (*P* < 0.05). No significant difference was found in the postoperative complications rate between the two groups (*P* > 0.05).

**Conclusion:** Granular bone grafts and transverse process bone grafts may achieve comparable clinical efficacy for single-segmental thoracic TB, but the former method had a shorter bone fusion time.

## Introduction

Spinal tuberculosis (TB) is the most common osteoarticular TB, which can cause vertebral body collapse, kyphosis deformity, and even compression of the spinal cord or nerve to cause paralysis in severe cases (1). Chemotherapy and surgery are the main treatment for spinal TB (2). The aim of the surgery is to relieve the compression of the spinal cord or nerve, reconstruct spinal stability, and correct kyphosis deformity (3, 4). Radical debridement of the TB lesion is the key to spinal TB surgery (5), but the vertebral defect is often left after the debridement, thus a bone graft is of great importance to restore the height of the vertebral body and rebuild the spinal stability (6). At present, the most commonly used bone graft methods in spinal TB surgery are iliac bone grafts and titanium mesh bone grafts (7), but the concerns of autologous bone donor site complications and titanium mesh subsidence get more and more attention (8, 9).

In recent years, it has been reported that granular bone grafts can achieve satisfactory clinical efficacy and safety in spinal TB surgery (10–12). Our previous study also found that granular bone grafts had a shorter operation time, less operation blood loss, and faster bone graft fusion than structural bone grafts for single-segmental thoracic TB (13). Moreover, we also proposed a new bone graft method in thoracic TB surgery, namely transverse process bone grafts. It was reported that transverse process bone grafts could obtain good clinical efficacy for single-segmental thoracic TB with an average bone graft fusion time of 5.85 months without serious complications (14). Besides, the transverse process bone graft was found superior to the iliac bone graft and titanium mesh bone graft in surgical trauma, postoperative recovery, and complications (15). Therefore, in our previous studies, both granular bone grafts and transverse process bone grafts showed satisfactory clinical results in thoracic TB surgery and thus have a good application prospect.

However, there was no study that compared the clinical efficacy of granular bone grafts and transverse process bone grafts in spinal TB surgery. Therefore, we designed this retrospective comparative study to evaluate the surgical efficacy of granular bone grafts and transverse process bone grafts in the surgical treatment of single-segmental thoracic TB.

## Methods

This retrospective single-center comparative study was approved by the Ethics Committee of the First Affiliated Hospital of Chongqing Medical University (No: 2017-067). Written informed consent to participate in this study was obtained for all the participants before their data were stored and used for research. This work has been reported in line with the STROCSS criteria (16).

### Patients Selection

Medical records of spinal TB patients who underwent surgery in our department from 2015 to 2018 were retrospectively analyzed.

#### Inclusion Criteria

(a) Thoracic TB (T1/2-T12/L1) confirmed by postoperative pathological examination. (b) Single-segmental thoracic TB with age >18 years. (c) Underwent one stage posterior debridement, bone graft fusion, and internal fixation. (d) Underwent granular bone graft or transverse process bone graft. (e) Follow-up time >12 months. (f) Clinical and imaging data were complete.

#### Exclusion Criteria

(a) Patients with spinal surgery history. (b) Patients with active pulmonary TB or malignant tumor, etc.

### Preoperative Management

X-ray, CT, and MRI examinations were taken for all patients, and preoperative sagittal Cobb angle was measured on lateral X-ray. Regular anti-TB chemotherapy (rifampicin 450 mg/d, isoniazid 300 mg/d, pyrazinamide 1,500 mg/d, and ethambutol 750 mg/d) was applied for all patients for at least 2–4 weeks before surgery. Surgery was taken when TB poisoning symptoms were relieved, the ESR returned to normal or had a significant decrease and basic diseases such as diabetes, coronary heart disease, and hypertension were under control.

### Surgical Procedures

**Figure 1:**
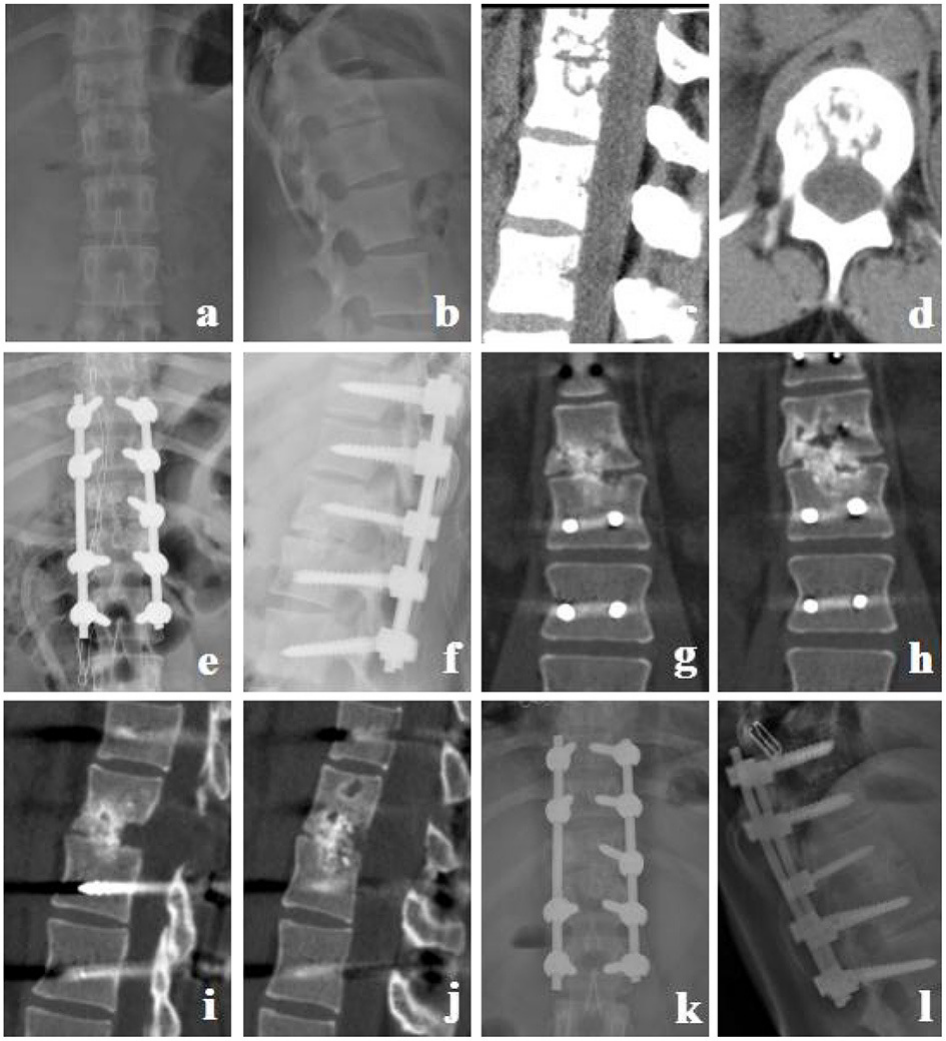
Granular bone graft group. A 22-year-old female with T12-L1 TB received a granular bone graft for reconstruction. **(a–d)** Preoperative X-ray and CT showed that the T12 and L1 vertebral bodies and the intervertebral disc were destroyed, and thoracolumbar instability was formed. **(e,f)** Postoperative X-ray. **(g–j)** CT at 5 months postoperative showed bone fusion between T12 and L1. **(k,l)** X-ray at 12 months postoperative showed good location of posterior instrument and normal thoracolumbar curve.

**Figure 2:**
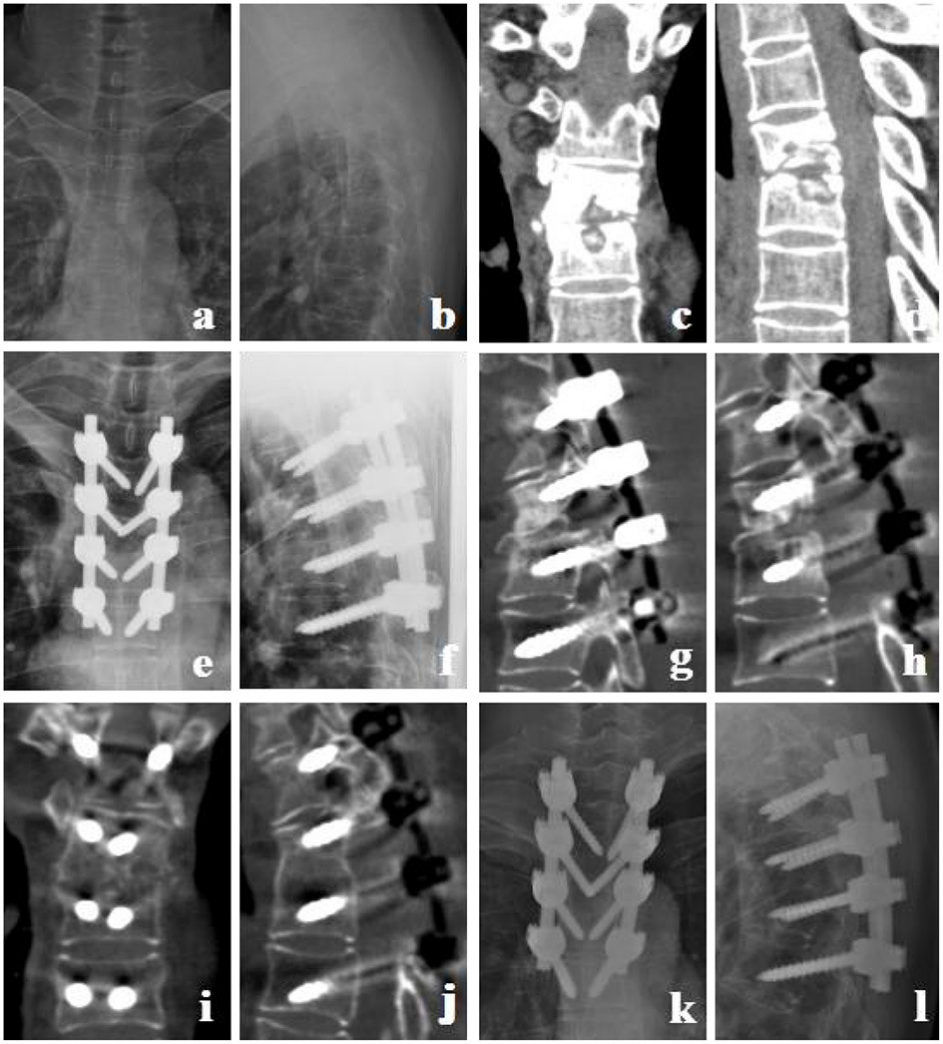
Transverse process bone graft group. A 43-year-old female with T4-5 TB received a transverse processes bone graft for reconstruction. **(a–d)** Preoperative X-ray and CT showed that T4 and T5 vertebral margins were irregularly destroyed and the intervertebral space narrowed. **(e,f)** Postoperative X-ray. **(g–j)** CT at 7 months postoperative showed bone fusion between T4 and T5. **(k,l)** X-ray at 12 months postoperative showed good location of posterior instrument and normal thoracic kyphosis.

The patient was placed in a prone position after general anesthesia, and a C-arm X-ray was used to locate the lesion segment. Bilateral paraspinal muscles were subperiosteal detached via a posterior median approach. For a unilateral TB lesion, an inter-muscular approach was applied as per our previous research (17). The spinous process, lamina, articular, and transverse process of the lesion segment, and the adjacent normal vertebrae were all exposed. Then pedicle screws were implanted into one or two normal vertebrae above and below the lesion segment and the titanium rod was temporarily locked. Bilateral vertebral plates were resected to decompress the spinal canal, and caseous necrosis, intervertebral disc, and dead bone were all completely stricken off by different types of curette. Then, the proper pressure was applied to the posterior screw system to correct kyphosis and a C-arm X-ray was used to confirm the kyphosis correction. Then, bone grafting was performed: (a) Granular bone graft: harvest the vertebral plate, spinous process, and articular process and make them into 3–5 mm granular bone. Then implant the granular bone into the intervertebral space and tamp them down. Finally, put a gelatin sponge containing isoniazid to cover the posterior margin of the granular bone to prevent them from entering the spinal canal (Figure 1). (b) Transverse processes bone graft: cut off one or two transverse processes of the adjacent segment and trim it to create a columnar cage with annular cortical bone on the sides and cancellous bone at both ends. Then implant it into the intervertebral space (Figure 2). Finally, place streptomycin 1.0 g and isoniazid 0.3 g in the TB lesion, place two drainage tubes in the incision, and then close the incision layer by layer.

### Postoperative Management

Antibiotics were used to prevent infection in the first 3 days postoperative. When drainage volume was <40 ml/d, the incision drainage was removed, and an X-ray examination was performed. After discharge, patients were requested to use a brace for 3 months and continue the anti-TB chemotherapy for 18–24 months. At 1, 3, 6, and 12 months postoperatively, the X-ray, ERS, CRP, hepatic and renal function, CT, and MRI (if necessary) were followed up. The sagittal Cobb angle was also measured on lateral X-ray.

### Outcome Indexes

#### Clinical Outcomes

(a) Operative time, operative blood loss, and hospital stay. (b) Visual analog scale (VAS) score, erythrocyte sedimentation rate (ESR), and C reactive protein (CRP). (c) The American Spinal Injury Association (ASIA) grade. (d) Complications.

#### Imaging Outcomes

(a) Cobb angle: the angle between the upper endplate of the upper vertebral body and the inferior endplate of the inferior vertebral body. (b) Bone graft fusion time: according to the CT scan bone graft fusion was evaluated by the criterion reported by Bridwell et al. (18). Grade I: Fused with remodeling and trabeculae. Grade II: Graft intact, not fully remodeled and incorporated though; no lucencies. Grade III: Graft intact, but a definite lucency at the top or bottom of the graft. Grade IV: Definitely not fused with resorption of the bone graft and with collapse. Grade I and Grade II were defined as bone graft fusion in this study.

### Statistical Analysis

Quantitative data were expressed in mean ± standard deviation. Inter-group and intra-group comparison of quantitative data were performed by independent sample *t*-test and matched *t*-test, respectively. Mann-Whitney rank sum test and Chi-square test were used for the inter-group comparison of ordered and disordered qualitative data, respectively. Statistical analysis was done by SPSS 19.0 software, and a significant difference was defined as *P* < 0.05.

## Results

A total of 52 patients were included with 25 cases in the granular bone graft group and 27 cases in the transverse processes bone graft group. No significant differences were found in age, gender, paravertebral cold abscess, and follow-up time between the two groups (Table 1).

**Table 1:** Comparison of preoperative clinical features between the two groups.

**Clinical features**	**Granular bone graft group** ** (*N* = 25)**	**Transverse process bone graft group** ** (*N* = 27)**	***P*-value**
Age (year)	39.7 ± 17.5	44.0 ± 14.8	0.347
Gender			0.137
Male	11	17	
Female	14	10	
Paravertebral abscess			0.511
Yes	16	19	
No	9	8	
Follow-up time (month)	28.1 ± 5.3	29.8 ± 4.8	0.431

There were no significant differences in operative time, operative blood loss, and hospital stay between the two groups. With an average follow-up of 14–33 months, the VAS scores, ESR, and CRP at the last follow-up were all significantly improved compared with preoperative measurements (*P* < 0.05), but no significant difference was found between the two groups (Table 2).

**Table 2:** Comparison of clinical outcomes between the two groups.

**Clinical outcomes**	**Granular bone graft group** ** (*N* = 25)**	**Transverse process bone graft group** ** (*N* = 27)**	***P*-value**
Operative time (min)	188.4 ± 47.2	206.3 ± 32.2	0.115
Operative blood loss (ml)	382.0 ± 258.2	461.1 ± 399.8	0.405
Hospital stay (day)	11.7 ± 4.3	13.4 ± 3.9	0.154
**VAS score**			
Preoperative	5.4 ± 1.1	5.5 ± 1.2	0.628
Last follow-up	1.7 ± 0.8^a^	1.4 ± 0.5^a^	0.065
**ESR (mm/h)**			
Preoperative	55.8 ± 25.3	55.7 ± 29.7	0.992
Last follow-up	14.1 ± 4.3^a^	13.0 ± 4.0^a^	0.370
**CRP (mg/L)**			
Preoperative	30.5 ± 28.7	29.6 ± 26.1	0.907
Last follow-up	6.5 ± 3.9^a^	6.9 ± 5.2^a^	0.785

*ESR, erythrocyte sedimentation rate; CRP, C-reactive protein*.

a *P < 0.05, compared with preoperative*.

In both the two groups, the postoperative Cobb angle was significantly corrected (*P* < 0.05), and showed a certain degree of loss during the follow-up (*P* < 0.05). There was no significant difference in Cobb angle correction between the two groups (*P* > 0.05). At the last follow-up, the granular bone graft group had a higher Cobb angle loss than the transverse processes bone graft group (*P* < 0.05). The bone graft fusion time in the granular bone graft group was significantly shorter than that of the transverse processes bone graft group (*P* < 0.05; Table 3).

**Table 3:** Comparison of imaging outcomes between the two groups.

**Imaging outcomes**	**Granular bone graft group** ** (*N* = 25)**	**Transverse process bone graft group** ** (*N* = 27)**	***P*-value**
Preoperative Cobb angle (°)	15.2 ± 11.3	15.2 ± 15.1	0.995
Postoperative Cobb angle (°)	5.0 ± 7.7	5.2 ± 12.6	0.938
Last follow-up Cobb angle (°)	6.5 ± 7.8	6.4 ± 12.6	0.997
Cobb angle correction (°)	10.2 ± 5.9	9.9 ± 7.8	0.896
Cobb angle loss (°)	1.5 ± 0.4	1.2 ± 0.3	0.001
Bone graft fusion time (month)	5.0 ± 0.9	6.3 ± 1.0	<0.001

Both the two groups achieved significant improvements in ASIA grade at the last follow-up (*P* < 0.05; Table 4). However, no significant difference was found in the last follow-up ASIA grade between them (*P* > 0.05).

**Table 4:** Comparison of neurological function (ASIA grade) between the two groups.

**Group**	**Preoperative ASIA**	**Postoperative ASIA**				
		**A**	**B**	**C**	**D**	**E**
Granular bone graft group (*N* = 25)	A	0	1	0	0	0
	B	0	0	0	0	0
	C	0	0	0	2	1
	D	0	0	0	0	4
	E	0	0	0	0	17
Transverse process bone graft group (*N* = 27)	A	0	0	0	0	0
	B	0	0	2	0	0
	C	0	0	0	1	1
	D	0	0	0	0	5
	E	0	0	0	0	18

A total of seven patients had postoperative complications in the transverse processes bone graft group with two cases of pulmonary infection, two cases of hepatic function damage, one case of renal function damage, and one case of sinus tract formation. While six cases of postoperative complications were found in the granular bone graft group with two cases of hepatic function damage, two cases of sinus tract formation, one case of pulmonary infection, and one case of urinary tract infection. There was no significant difference in complications between the two groups (*P* > 0.05), and all cases were cured after active treatment.

## Discussion

To our knowledge, there are only a few reports of transverse processes bone grafts for repairing spinal TB bone defects so far. Anatomical studies show that the average length of the transverse process of the thoracic vertebra is 17.4 mm, thoracic vertebral height is 14.1–22.7 mm (19), and the height of the thoracic intervertebral disc is 2–6 mm (20). So when the destruction of the two adjacent vertebral bones is less than half of the vertebral body height, a transverse processes bone graft can meet the needs. Moreover, Thanapipatsiri and Chan (21) studied the relationship of the transverse process and its adjacent structures (including local blood vessels, nerve) and concluded that no important blood vessels, nerves, muscles, and tendons would be injured during the transverse process harvesting as long as the subperiosteal dissection was strictly followed with gentle movements. Located in the surgical segments of TB debridement and internal fixation, transverse processes are easy to expose and harvest and has little effect on spinal stability after resection (22), and thus radical debridement, decompression, bone grafts, and internal fixation can be performed in only one incision, which reduces surgical trauma and shortens the operative time (14, 15).

Previous studies found that both granular bone grafts and transverse process bone grafts have the advantages of shorter operation time and less intraoperative bleeding compared with iliac bone grafts and titanium mesh bone grafts (13, 15). But no differences were found in operative time, operative blood loss, and hospital stay between the granular bone graft and transverse process bone graft. We thought this may be due to the following reasons: (a) Although the decompression, bone graft, and internal fixation could be done in one incision in the transverse process bone graft group, the bone grafting beds needed to be prepared (14) and the harvested transverse process bone had to be trimmed to fit the size of the intervertebral space defect (15). (b) Although the granular bone is small and convenient to implant and does not have a high requirement for the condition of a bone grafting bed (12), surgeons still need to crush the lamina, and undergo the spinous process and articular process to make bone pellets (13). (c) Similar surgical trauma of the two groups may lead to no difference in the hospital stay.

This study found the bone graft fusion time of the granular bone graft was shorter than the transverse process bone graft, which may be related to the promoting osteogenesis effect of granular bone for the following reasons: (a) granular bone has a large contact area with the vertebral body and it is conducive for nutrient infiltrating and neovascularization growth (23). (b) Granular bone can induce surrounding bone mesenchymal cell proliferation and secretion of bone morphogenetic protein (BMP), thus promote osteogenesis (24). (c) Granular bone squeezes with each other, deformation and local stress can stimulate bone growth (25).

In this study, we also found that the Cobb angle loss of the granular bone graft group was more obvious than that of the transverse process bone graft group during the follow-up, which may due to the potential disadvantages of granular bone such as smaller diameter, weak supporting force (10, 11), and ease in which it can be completely absorbed (13). Although a granular bone graft is prone to Cobb angle loss after surgery, the loss was slight (<3°), and our previous study already has concluded that mild Cobb angle loss after STB surgery did not affect bone graft fusion, spinal stability, and clinical symptoms (13, 26). There was no significant difference between the granular bone graft and transverse bone graft in alleviating clinical symptoms and complications, and this may indirectly confirm the above views.

At the last follow-up, the VAS score, ESR, CRP, and ASIA grade of the two groups were all significantly improved compared with preoperative measurements, this is due to the following reasons: (a) All patients received long-term and effective anti-TB chemotherapy (27). (b) Radical debridement of the TB lesion, effective decompression of spinal canal, correction of kyphosis, and rigid posterior internal fixation during the surgery (28, 29). (c) Bone graft fusion was obtained in all patients at the last follow-up. In this study, the postoperative complications after spinal TB mainly included hepatic function damage, sinus formation, and pulmonary infection, which may be due to the long-term anti-TB chemotherapy, long hospital stay, and low immunity of TB patients. However, no difference in complication rate was found between the two groups, and all the complications were recovered through active treatment. This also indicated that both the granular bone graft and transverse process bone graft were safe in thoracic spinal TB surgery.

In our opinion, the granular bone graft and transverse process bone graft have similar indications for single-segmental thoracic TB (13). The indications were as follows: (a) Deterioration of neurological dysfunction or paralysis. (b) Progressive spinal instability or kyphosis deformity. (c) Single-segmental thoracic TB with vertebrae destruction <50% of the vertebrae height. (d) The TB lesion was mainly in the former column and the posterior column was not involved. Transverse process bone grafts may be a good alternative method when the volume of granular bone is not enough to meet the requirements of intervertebral bone grafting in spinal TB surgery.

Our study also had some limitations. Firstly, this was a single-center retrospective study with a small sample size. Secondly, the follow-up time was short. Third, different level surgeons may have different experiences in the two bone graft methods.

## Conclusion

A granular bone graft has a shorter bone graft fusion time compared with a transverse process bone graft in one stage posterior debridement, bone graft fusion, and internal fixation for single-segmental thoracic TB. The two methods may both achieve satisfactory clinical efficacy in appropriate cases. Due to the potential limitations, prospective randomized studies with a large sample size and long follow-up period are needed to validate our findings.

## Data Availability Statement

The raw data supporting the conclusions of this article will be made available by the authors, without undue reservation.

## Ethics Statement

The studies involving human participants were reviewed and approved by the Ethics Committee of the First Affiliated Hospital of Chongqing Medical University. Written informed consent to participate in this study was provided by the participants' legal guardian/next of kin.

## Author Contributions

XD and YO contributed to the study design. XD, YO, and YZ performed the surgery. XD, WL, and GJ collected the data. XD, YZ, and DJ analyzed the data. XD and GJ wrote the manuscript. All authors read and approved the final manuscript.

## Conflict of Interest

The authors declare that the research was conducted in the absence of any commercial or financial relationships that could be construed as a potential conflict of interest.
